# Mouse DNA contamination in human tissue tested for XMRV

**DOI:** 10.1186/1742-4690-7-108

**Published:** 2010-12-20

**Authors:** Mark J Robinson, Otto W Erlwein, Steve Kaye, Jonathan Weber, Oya Cingoz, Anup Patel, Marjorie M Walker, Wun-Jae Kim, Mongkol Uiprasertkul, John M Coffin, Myra O McClure

**Affiliations:** 1 Section of Infectious Diseases, Jefferiss Research Trust Laboratories, Imperial College London, St Mary's Campus, London, W2 1PG, UK; 2 Department of Molecular Biology & Microbiology and Program in Genetics, Tufts University, Boston, MA, USA; 3 Department of Urology, Imperial College Healthcare NHS Trust, St Mary's Hospital, London, W2 1PG, UK; 4 Department of Histopathology, Imperial College London, St Mary's Hospital, London, W2 1PG, UK; 5 Department of Urology, College of Medicine, Personalised Tumor Engineering Research Centre, Chungbuk National University, Chungbuk 361-763, Korea; 6 Department of Pathology, Faculty of Medicine, Siriraj Hospital, Mahidol University, Bangkok, 10700, Thailand

## Abstract

**Background:**

We used a PCR-based approach to study the prevalence of genetic sequences related to a gammaretrovirus, xenotropic murine leukemia virus-related virus, XMRV, in human prostate cancer. This virus has been identified in the US in prostate cancer patients and in those with chronic fatigue syndrome. However, with the exception of two patients in Germany, XMRV has not been identified in prostate cancer tissue in Europe. Most putative associations of new or old human retroviruses with diseases have turned out to be due to contamination. We have looked for XMRV sequences in DNA extracted from formalin-fixed paraffin- embedded prostate tissues. To control for contamination, PCR assays to detect either mouse mitochondrial DNA (mtDNA) or intracisternal A particle (IAP) long terminal repeat DNA were run on all samples, owing to their very high copy number in mouse cells.

**Results:**

In general agreement with the US prevalence, XMRV-like sequences were found in 4.8% of prostate cancers. However, these were also positive, as were 21.5% of XMRV-negative cases, for IAP sequences, and many, but not all were positive for mtDNA sequences.

**Conclusions:**

These results show that contamination with mouse DNA is widespread and detectable by the highly sensitive IAP assay, but not always with less sensitive assays, such as murine mtDNA PCR. This study highlights the ubiquitous presence of mouse DNA in laboratory specimens and offers a means of rigorous validation for future studies of murine retroviruses in human disease.

## Background

In 2006, XMRV was identified in stromal cells associated with prostate cancers of men with a family history of the malignancy, 40% of whom were homozygous for a specific variant of the interferon-inducible RNaseL gene, suggesting an increased susceptibility to viral infection in these patients. Virochip microarray analysis on cDNA from some of these tumours led to the identification of XMRV [1], a gammaretrovirus closely related, but not identical, to endogenous MLV. Interest in XMRV intensified when 6% of all prostate cancers in a US clinic were found to carry the virus and when by immuno-histochemical staining the virus was detected in the tumour epithelium of 23% of those patients. In the latter study, virus detection was associated with a higher Gleason Index and appeared to be independent of the RNAseL mutation [2]. Several papers have since demonstrated a link of prostate cancer with XMRV [3-5], however this has not been repeated in other cohorts [6-9].

XMRV has also been reported in patients suffering from chronic fatigue syndrome (CFS) [10], a condition also associated with perturbations in the RNaseL innate defence response. In addition, XMRV has been described in the bronchiolar lavages of immunosuppressed individuals [11]. Several groups have presented unambiguous data challenging the original findings, both in European CFS cohorts [12-14] and in US CFS patients [15,16]. More recently, matters were further complicated by the publication of a study finding *gag *sequences similar to those of four different polytropic endogenous MLVs in an unrelated US CFS cohort, but no evidence of XMRV [17]. A clear account of the claims and counter-claims surrounding XMRV and its disease association has recently been published [18].

## Methods

The UK prostate specimens in this study came from two sources. Cancer tissue came from men locally referred to St Mary's Hospital in West London with symptoms of voiding dysfunction and prostate specific antigen abnormality and requiring biopsy after appropriate counselling. In addition, some patients had participated in a voluntary screening study and these provided samples that were a mixture of benign and cancer pathologies. All biopsy tissue had been stored in formalin fixed, paraffin-embedded (FFPE) blocks over a period of 3-6 years. Slices were taken from the blocks and DNA extracted as described (see Additional File 1; Supplementary Methods). The FFPE samples from Thailand and Korea were excess tissue from histological samples taken from new cases of both benign prostate hyperplasia and prostate cancer. These were FFPE embedded and sent to London for analysis.

We investigated the prevalence of XMRV in the UK and in the Far East, aware that the close relationship (about 94% at the nucleotide level) to other murine exogenous and endogenous retroviruses posed a problem in distinguishing XMRV from contaminating mouse DNA sequences. We were further aware that in any retrovirology laboratory MLV sequence contamination is something of an occupational hazard [19]. For these reasons, we extracted the DNA from FFPE prostate cancers, along with benign hyperplasia tissue, and PBS without tissue. We used several sets of primers [12] to test for XMRV-specific sequences, derived from the XMRV *gag *leader [1] which encompasses the 24 bp deletion originally thought to distinguish XMRV as a new human virus. To control for low level contamination, we included multiple no-template controls (no less than 6 in every run) and included assays with primers that would amplify murine mitochondrial DNA (mtDNA) and intracisternal A particle (IAP) LTRs. IAPs are retrotransposons present at the level of about 1000 copies of varying length per mouse genome [20].

## Results

All murine retroviral primer sequences amplified specific products of the appropriate size when tested on pXMRV isolate VP62, an infectious molecular clone of XMRV, kindly provided by R. Silverman. The IAP primers did not amplify sequences from human DNA extracted from LNCaP cells (prostate cancer cell line) or from six PBMC samples from human prostate cancer patients (data not shown).

**Figure 1:**
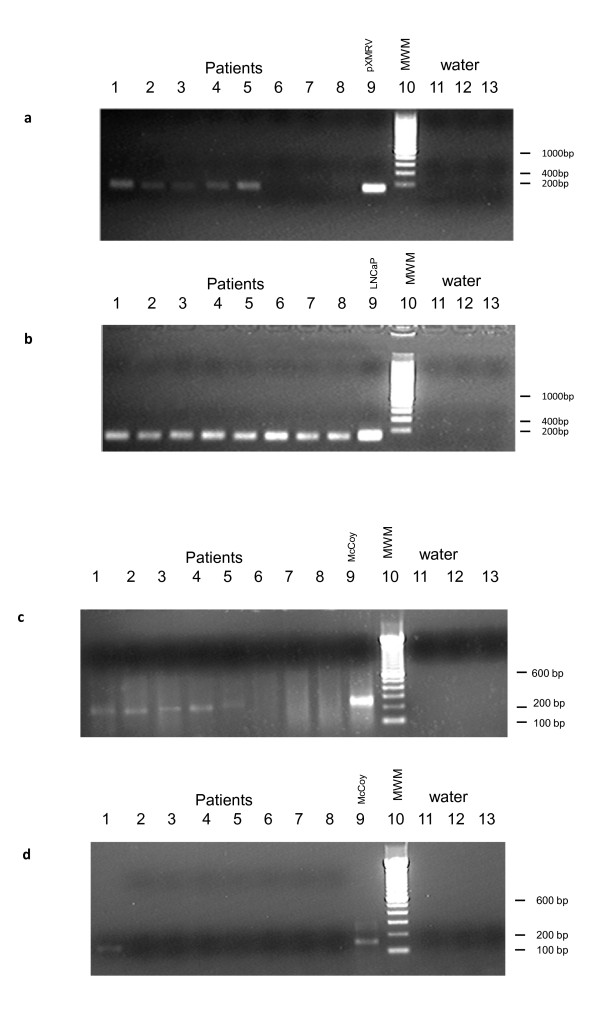
**Nested PCR on DNA extracted from FFPE tissue of prostate cancer patients**. Figure (a) shows samples that produced a PCR product of the expected size using primers specific for XMRV (lanes 1-5). UK patient 308 and UK 244 (Lane 1 and 2); Thailand 1 and Thailand 2 (Lane 3 and Lane 4); Korea 62 (Lane 5). Lanes 6-8 show samples in which XMRV was not detected. Thailand 3 (Lane 6); Korea 60 (Lane 7); Korea 61(Lane 8); Positive control pXMRV produces a strong band (lane 9). Promega 200 bp DNA step ladder (lane 10). Lanes 11-13 show water negative controls. Figure (b) shows β-globin control PCR used to demonstrate the presence of human DNA in each sample (lanes 1-8, as above). Expected size was 104 bp. Positive control LNCaP DNA is shown in lane 9. Lanes 11-13 show negative water controls. All patient samples tested showed a positive signal for β-globin. Figure (c) shows the IAP PCR result for the same samples (lanes 1-5 IAP positive, lanes 6-8 IAP negative). Positive control McCoy cell DNA is shown in lane 9. Invitrogen 100 bp DNA step ladder (lane 10). Lanes 11-13 show negative water controls. Figure (d) shows the mtDNA PCR results for the same sample (lane 1 mtDNA positive, lanes 2-8 mtDNA negative) Positive control McCoy cell DNA is shown in lane 9. Invitrogen 100 bp DNA step ladder (lane 10). Lanes 11-13 show negative water controls.

FFPE prostate tumour slices (two 10 micron slices from each lobe of the prostate) were provided to us blinded by the Histopathology Department at St Mary's Hospital in batches and randomised with benign prostate hyperplasia specimens. For samples received from Thailand and Korea, those carrying out the PCR were blinded to sample provenance. In all cases, care was taken to use a fresh blade for slicing each patient sample, and the top slice was discarded. In total, of 292 UK prostate cancers analysed, 14 were XMRV-positive by PCR using the *gag *leader primers, as were five out of 139 Korean samples and two out of six from Thailand. A representative result is shown in Figure 1.

All FFPE prostate cancer specimens from the Pathology Department at St Mary's hospital London were provided to us in batches. The tissue slices were coded and assayed blind. Initially, the PCR amplification and sequence analysis of the amplicons encouraged us to deduce that we had detected a genuine XMRV infection in some of the samples. When on unblinding we found a concordant result from the same patient whose duplicate specimens had been provided in different batches, this appeared to reaffirm a genuine XMRV infection. Moreover, in two patients in whom the tumour was unilateral, XMRV was detected only in the cancerous lobe. Taken together with the consistently negative PCR water controls, the probability of contamination appeared to be low.

**Figure 2:**
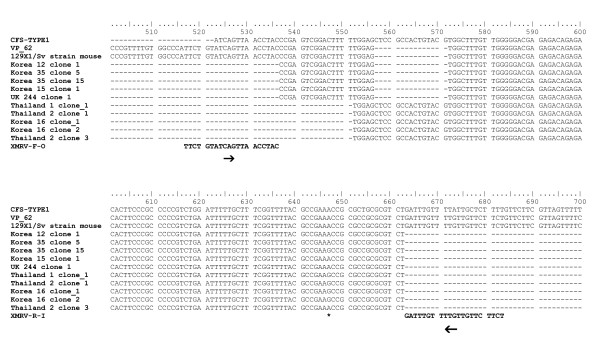
**Sequence alignment of XMRV LTR from 7 prostate cancer patients**. The *gag *leader primer set XMRV-R-I/XMRV-F-O bind either side of the XMRV specific deletion. Sequences were aligned against VP62 [Genbank:EF185282], MLV-releated virus CFS isolate CSF-type1 ([Genbank:HM630562], as described in Lo [17]) and mouse strain 129X1/SvJ [Genbank AAHY01591888.1]. The alignment was conducted with clustalW using BioEdit v7.0.5.3. Binding sites of primers XRMV-R-I and XMRV-F-O are shown. The asterisk shows the location of the A > G mutation.

Upon sequencing, however, we noticed differences in the obtained PCR products. Some sequences displayed the deletion characteristic of XMRV upstream of the *gag *ATG (Korean samples 12, 35, 15), and UK (sample 244), while others did not (Thai patients 1 and 2 and Korean 16) (Figure 2). Downstream of this deletion all sequences are identical, apart from an A > G mutation at position 647, but there is no correlation of the A647G mutation with the presence of the deletion. Sequences that did not contain the deletion were amplified, indicating that the primers were not as specific to XMRV as expected. A BLAST search showed the best match for sequences without the deletion but containing a G at position 647 to be mouse endogenous polytropic provirus clone 15 [Genbank:FJ544577.1], which suggests the presence of mouse DNA in these samples. It has been shown recently that the 24 bp deletion specific to XMRV [1], is also present in the sequence of a polytropic endogenous MLV sequence [Genbank:AAHY01591888.1] in the laboratory mouse strain 129X1/SvJ, commonly used in gene knock-out experiments [21]. Our sequences (Korean samples 12, 35, 15; and UK sample 244) are identical to this PMLV sequence present in strain 129X1/SvJ (Figure 2). We therefore, sought to investigate this potential contamination further.

The IAP and mtDNA PCR assays were applied to 10-fold dilution series of McCoy cell and RAW 264.7 cell DNA to compare the sensitivity of the methods for detection of genomic mouse DNA. We found the mtDNA PCR (14) 100-fold less sensitive than that for IAP in both cell lines (see Additional File 2; Figure S1). The IAP PCR, thus, provided a far more reliable indicator of contaminating murine sequences. The IAP and mtDNA PCR assays were applied to the same sample to test whether the apparent XMRV positivity might have been due to mouse DNA contamination. Amplification of XMRV-specific sequences was completely concordant with amplification of IAP sequences from the same DNA (Table 1). Samples from 292 UK patients, of which 212 (73%) were cancerous, 68 (23%) were benign and 12 (4%) were lost to follow up, along with 139 Korean samples (all cancerous) and 6 Thai samples (50% cancerous, 50% benign) were tested by XMRV, IAP and mtDNA by PCR. Twenty-one samples were positive for XMRV using the *gag *leader primers, and of these, 17 were from cancerous tissue and 4 from benign tissue. Overall, 115/437 (26.3%) of the samples, including all 21 of the XMRV-positives were positive for IAP sequences and 21/115 (18.2%), of the IAP positives contained mouse mtDNA. To confirm that the sequences amplified by the IAP primers were indeed murine in origin, we cloned and sequenced one of the amplicons. Several IAP sequences were obtained (see Additional File 3; Figure S2). The fact that not all IAP positives were XMRV positive may be explained by the low level of contamination of murine DNA that would contain only a few copies of endogenous XMRV like sequences compared to the many IAP copies per genome.

**Table 1:** Frequency of positive PCR reactions using XMRV LTR primers, mtDNA primers and IAP primers.

	XMRV +	XMRV -
**IAP +** **mtDNA +**	**21** **(1^†^)**	94 (20)

IAP - mtDNA -	**0** **(5)**	322 (394*)

292 UK, 139 Korean, and 6 Thai samples were tested for XMRV, IAP and mtDNA sequences by PCR. ^†^13 not done, *4 not done due to lack of sample

## Discussion

XMRV shares extensive sequence identity with known xenotropic, nonecotropic and polytropic murine viruses; the first of which is known to infect many common human tumour cell lines, a phenomenon that has confused retrovirologists looking for disease associations for over three decades. Most putative associations of new or old human retroviruses with diseases (including CFS and prostate cancer) have turned out to be laboratory artefacts [19]. The case of XMRV as a new human pathogen must be judged against this background [22]. It is true that we cannot formally rule out the possibility that the samples in question are infected with XMRV and simultaneously contaminated by mouse DNA, although this is unlikely since we found no IAP-negative samples from which we amplified MLV-specific sequences (data not shown). Also, the failure to detect XMRV sequences other than in association with mouse DNA contamination in our cohort does not mean that the virus is not present in other, unrelated, cohorts.

It is difficult to explain how the contamination may have occurred, especially since the samples came from three unrelated centres in the UK, Korea and Thailand. As both our negative tissue and PBS controls treated in parallel with the FFPE were XMRV PCR-negative, it is unlikely that contamination was introduced via reagents. The UK FFPE tissue samples were stored boxed and stacked in a cupboard in the histopathology department for several years; and it is possible that contamination happened during that time, although why only a few samples (4.8%) were XMRV positive and the remainder not is difficult to explain. Nor does it explain the Thai and Korean results on tissue collected prospectively for the study. It does, however, demonstrate the necessity of controlling by highly specific and sensitive means for mouse contamination.

## Competing interests

The authors declare that they have no competing interests.

## Authors' contributions

MR, OE, SK carried out the experiments. MR & OE contributed equally. AP provided prostate tissue and clinical expertise. MW provided biopsy sections and pathology expertise. W-J K and MU provided prostate tissue from Korea and Thailand, respectively. OC and JC designed IAP assay. MM directed research and wrote the manuscript. All authors read and approved the final manuscript.

## Supplementary Material

Additional file 1 **Supplementary Methods**. Materials and Methods. Click here for file

Additional file 2 **Figure S1**. Sensitivity of mitochondria and IAP PCRs. Click here for file

Additional file 3 **Figure S2**. Sequence alignment of IAP PCR products. Click here for file
