# Supplementary material for: Mouse DNA contamination in human tissue tested for XMRV
Source: Retrovirology. 2010 Dec 20;7:108. doi: 10.1186/1742-4690-7-108 (PMC3019155; doi:10.1186/1742-4690-7-108)
Supplement: Additional file 1 — Supplementary Methods. Materials and Methods. [file 1742-4690-7-108-S1.DOC]

**Supplementary Information**

**Materials and Methods**

**Patients**

In 1998, ethical approval was sought and obtained to establish at St Mary’s Hospital, London, a prostate cancer tissue bank for freshly harvested operating room surgical specimens from men undergoing radical retro-pubic prostatectomy for clinically localized prostate cancer (PSA range 2.2ng/ml to 33ng/ml, median age 62 years). All patients gave written informed consent for their tissue to be banked for the purposes of research. The Tissue Bank ethics number 98CC141: A study to collect prostate tissue & bone marrow specimens to help establish methods for diagnosing and treating prostatic cancer.

The prostate with attached seminal vesicles and ampullary vasal stumps were conveyed dry immediately after release from the pelvis to the pathology lab, where apical, peripheral and bladder neck blocks were freshly sectioned by a waiting technician for pathological evaluation of surgical margin status later (after fixation and embedding in paraffin blocks), or intra-operative frozen section to check inked margin status at the site of neurovascular bundle sparing. The remaining central material was flash frozen and placed in the tissue bank and stored at -70C. The patient set studied was a well-characterised and representative sample of PC patients, referred within the UK National Health Service and were all routine attendees at the Prostate Assessment and Counselling for Treatment (Pro-ACT) Clinic at St Mary’s Hospital, London, under the care of one consultant urological surgeon (AP).

In addition, prostate biopsy cores obtained from a prostate cancer screening study (ethical LREC code 99/CC/166) from men aged 50-65 years in a community population registered with 10 local general practitioners were also available for further study from paraffin blocks. Men in this study were eligible for transrectal ultrasound -guided prostate needle biopsy if PSA was found to be >4ng/ml or between 1-3ng/ml with a low % free PSA of <20%. Of 3562 letters of invitation to screening sent at that time, 775 replied expressing an interest to be screened and after counselling, 2 men declined further participation, leaving 773 men (mean age 57.7 years), majority Caucasian (78.9%), with 10% Afrocaribbean, 5% Asian, 3.4% Middle eastern, and 1.8% Oriental. Of these men, 66 had PSA =/>4ng/ml (56 4-10ng/ml), 60 agreed to have biopsy and cancer detection rate was 33%; 355 had PSA 1.1-3.99ng/ml and 137 men from this second cohort had %free PSA =/< 20%, with an 11.3% positive biopsy rate.

Prostate cancer biopsy samples were also obtained from Dr Wun-Jae Kim of Chungbuk National University, Chungbuk, Korea and from Dr Mongkol Uiprasertkul of Siriraj Hospital, Bangkok, Thailand.

**PCR amplification of XMRV**

DNA was extracted from two 10 micron sections of the FFPE sample using the QIAamp DNA FFPE Tissue Kit (Qiagen, Crawley, UK), according to the manufacturer’s instructions. DNA was eluted in a volume of 50µl The range of input DNA was 145-700ng per reaction. To detect XMRV-specific PCR products nested PCR was used and for sequencing purposes, a semi-nested PCR was employed.

The conditions for the nested PCR and the sequences of the primers specific for the XMRV infectious molecular clone, VP62, as well as the generic primers used to detect MLV sequences and the primers for the detection of beta-globin have all been described elsewhere (Erlwein et al. 2010 [12]). For the sake of clarity, the primers XMRV forward outer, XMRV reverse outer, XMRV forward inner and XMRV reverse inner are referred to as XMRV-F-O, XMRV-R-O, XMRV-F-I and XMRV-R-I, respectively. The protocol for the semi-nested PCR included transferring of 5 µl of the first round into the second round instead of 1ul and using primer pair XMRV-F-O and XMRV-R-I instead of XMRV-F-I and XMRV-R-I. The products were visualised on a 2% Ethidium Bromide stained agarose gel. We have previously demonstrated the sensitivity of this PCR to detect one copy of plasmid DNA in a background of 500ng non-specific DNA For routine experiments, 0.11ng of plasmid DNA was used as a positive control

**PCR amplification of human beta globin**

Amplification of a section of the human beta globin gene has been described by Erlwein *et al*[12]. Briefly, a nested PCR was performed using primers to amplify a 124bp fragment of the human beta globin gene as a control for DNA integrity. LNCaP DNA was used as a positive control. Products were visualised on a 2% Ethidium Bromide stained agarose gel.

**PCR amplification of murine mitochondrial DNA**

Amplification of the mouse mitochondrial gene was carried out as described by Lo *et al* [17]. Briefly, a semi-nested PCR using primers to conserved sequences of mouse mitochondria. Reactions were in a volume of 25µl containing 0.5 units TaqGold polymerase (Applied BioSystems, Warrington, UK), 1xTaqGold reaction buffer (Applied BioSystems), 1.5mM Mg^2+^, 2.5 pmol forward primer MM1 (AGA CGC ACC TAC GGT GAA GA), 2.5 pmol reverse primer MM2 (AGA GTT TTG GTT CAC GGA ACA TGA) and 200mM each dNTP. The sample was 2µl extracted DNA. Second round reaction used 2.5 pmol forward primer MM3 (TGC CAA ACC CCA AAA ACA CT) and 2.5 pmol reverse primer MM2. Reaction conditions were one cycle of 94^o^C, 2 minutes, 30 cycles of 94^o^C 30 seconds, 58^o^C 30 seconds, 72^o^C 45 seconds and one cycle of 72^o^C, 7 minutes. The same reaction conditions were repeated for the second round. Products were visualised on a 2% Ethidium Bromide stained agarose gel.

**PCR amplification of IAP sequences**

Amplification of the IAP sequences of the mouse genome was carried out in a single round of PCR using primers to conserved IAP sequences. Reactions were in a volume of 25µl containing 0.5 units TaqGold polymerase (Applied BioSystems, Warrington, UK), 1xTaqGold reaction buffer (Applied BioSystems), 1.5mM Mg^2+^, 2.5 pmol forward primer (ATA ATC TGC GCA TGA GCC AAG G), 2.5 pmol reverse primer (AGG AAG AAC ACC ACA GAC CAG A) and 200mM each dNTP. The sample was 2µl extracted DNA. Reaction conditions were one cycle of 94^o^C, 8 minutes, 35 cycles of 94^o^C 30 seconds, 58^o^C 30 seconds, 72^o^C 20 seconds and one cycle of 72^o^C, 7 minutes. Products, of variable size, reflecting diversity of the IAP element, were visualised on a 2% Ethidium Bromide stained agarose gel.

**Assay for mtDNA and IAP PCR sensitivity on murine cell lines**

McCoy mouse fibroblast cells (ECACC #90010305) and RAW 264.7 mouse monocyte macrophage cells (ECACC #91062702) were grown in DMEM supplemented with 10% FCS and 1% penicillin/streptomycin at 37^o^C in a humidified atmosphere containing 5% CO_2_. DNA of 1x10^6^ cells was extracted using a Qiagen QIAamp DNA mini kit according to the manufacturer’s instructions. 150ng of DNA was diluted ten-fold in water to a final concentration of 1.5x10^-4^ pg, and each reaction was spiked with 500ng of LNCaP DNA. Reaction mixes were subjected to PCR as described above.

**Sequence analysis of IAP amplicon**

To confirm that the IAP PCR was amplifying murine IAP sequences the amplicon from one sample (UK patient 120) was cloned into the TOPO-TA vector (Invitrogen, Paisley, UK) according to the manufacturer’s instructions. Plasmid DNA was extracted from 11 positive clones and sequenced using primers targeted to the T3 and T7 sites within the vector. Sequences are provided in Figure S1.

**Figure S1| sensitivity of mitochondria and IAP PCRs.**

10-fold dilutions of murine cell genomic DNA were analysed using both mtDNA and IAP PCRs (lanes 1-10). McCoy DNA concentration was 110µg.µl^‑1^ and 1µl DNA was added per reaction. No-template controls are shown in lanes 11-14. Figure (a) mtDNA PCR which produces a product with 0.11pg input DNA (lane 9). Figure (b) IAP PCR, which produces a product with 0.0011pg input DNA (lane 7), indicating IAP was 100x more sensitive than mtDNA PCR. The experiment was repeated using RAW 267.4 cells (Figures c and d). DNA concentration was 173µg.µl^‑1^ and 1µl DNA was added per reaction. Lanes 1-11 show the dilution series 1 to 1:10^10^. Lanes 12-16 show no-tempate controls. Figure (c) IAP PCR, produces a product with 0.0173pg input DNA (lane 8). Figure (d) mtDNA PCR, produces a product with 1.73pg input DNA (lane 6), confirming the 100x sensitivity of IAP PCR compared to mtDNA.

**Figure S2| Sequence alignment of IAP PCR products.**

IAP sequences from UK patient 120 were obtained by TOPO cloning as described in Materials and Methods (see Additional File 1; Supplementary Methods). Clones 3, 5, 7, 10, 11, 13, 15, 14, 18, 19 and 20 were sequenced and aligned against IAP from chromosome 17 , genbank accession number NT039649.7 (labelled IAP chromo). The alignment was conducted with clustalW using BioEdit v7.0.5.3. BLAST search of the IAP sequence matched 100% to numerous mouse sequences.
