# Supplementary material for: Mouse DNA contamination in human tissue tested for XMRV
Source: Retrovirology. 2010 Dec 20;7:108. doi: 10.1186/1742-4690-7-108 (PMC3019155; doi:10.1186/1742-4690-7-108)
Supplement: Additional file 3 — Figure S2. Sequence alignment of IAP PCR products. [file 1742-4690-7-108-S3.DOC]

....|....| ....|....| ....|....| ....|....| ....|....| ....|....| ....|....| ....|....| ....|....| ....|....|

10 20 30 40 50 60 70 80 90 100

**IAP_chromosome 17**  GGTATCTTAT GACTACTTGT GCTCTGCCTT CCCCGTGA-C GTCAACTCGG CCGATGGGCT GCAGCCAATC AGGGAGTGAC ACGTCCGAGG CGAAGGAGAA

**IAP_3_180810**  GGTATCTTAT GACTACTTGT GCTCTGCCTT CCCCGTGA-C GTCAACTCGG CCGATGGGCT GCAGCCAATC AGGGAGTGAC ACGTCCGAGG CGAARGAGAA

**IAP_5_180810**  GGTATCTTAT GACTACTTGT GCTCTGCCTT CCCCGTGA-C GTCAACTCGG CCGATGGGCT GCAGCCAATC AAGGAGTGAC ACGTCCGAGG CGAAGGAGAA

**IAP_7_180810**  GGTATCTTAT GACTACTTGT GCTTTGCCTT CCCCGTGA-C GTCAACTCGG CCGATGGGCT GCAGCCAATC AGGGAGTGAC ACGTCCGAGG CGAAGGAGAA

**IAP_10_180810**  GGTATCTTAT GACTACTTGT GCTCTGCCTT CCCCGTGA-C GTCAACTCGG CCGATGGGCT GCAGCCAATC AGGGAGTGAC ACGTCCGAGG CGAAGGAGAA

**IAP_11_180810**  GGTAT-TTAC GACTACTTGT ACTCTGTTTT TCCCGTGAAC GTCAGCTCGG CC-ATGGGCT GCAGCCAATC AGGGAGTGAT GCGTCCTAGG CAATTGTTGT

**IAP_13_0180810**  GGTATCTTAT GACTACTTGT GCTCTGCCTT CCCCGTGA-C GTCAACTCGG CCGATGGGCT GCAGCCAATC AGGGAGTGAC ACGTCCGAGG CGAAGGAGAA

**IAP_14_180810**  GGTATCTTAT GACTACTTGT GCTCTGCCTT CCCCGTGA-C GTCAACTCGG CCGATGGGCT GCAGCCAATC AGGGAGTGAC ACGTCCGAGG CGAAGGAGAA

**IAP_15_180810**  GGTATCTTAT GACTACTTGT GCTCTGCCTT CCCCGTGA-C GTCAACTCGG CCGATGGGCT GCAGCCAATC AGGGAGTGAC ACGTCCGAGG CGAAGGAGAA

**IAP_18_180810**  GGTATCTTAT GACTACTTGT GCTCTGCCTT CCCCGTGA-C GTCAACTCGG CCGATGGGCT GCAGCCAATC AGGGAGTGAC ACGTCCGAGG CGAAGGAGAA

**IAP_19_180810**  GGTATCTTAT GACTACTTGT GCTCTGCCTT CCCCGTGA-C GTCAACTCGG CCGATGGGCT GCAGCCAATC AAGGAGTGAC ACGTCCGAGG CGAAGGAGAA

**IAP_20_180810**  GGTATCTTAT GACTACTTGT GCTCTGCCTT CCCCGTGA-C GTCAACTCGG CCGATGGGCT GCAGCCAATC AAGGAGTGAC ACGTCCGAGG CGAAGGAGAA

....|....| ....|....| ....|....| ....|....| ....|....| ....|....| ....|....| ....|....| ....|....| ....|....|

110 120 130 140 150 160 170 180 190 200

**IAP_chromosome 17**  TGCTCCTTAA GAGGGACGGG GTTT-CGTTT TCTCTCTCTC TTGCTTCTCG CTCTCTCTTG CTTC------ -----TTGCT CTCTTGCTT- -CTTACACTC

**IAP_3_180810**  TGCTCCTTAA GAGGGACGGG GTTT-CG--T TYTCTCTCTC TYTTGCTTCT YKCKCTCTTG CTTC------ -----TTGCT CTCTTGCTT- -CTTACACGC

**IAP_5_180810**  TGCTCCTTAA GAGGGACGGG GTTT-CGTTT TCTCTCTCTC TTGCCTCT-- --CTCTCTTG CTTT------ ---TCTCTCT CTCTTGCTT- -CTTGCTCTC

**IAP_7_180810**  TGCTCCTTAA GAGGGACGGG GTTT-CG--T TTTCTCK--- ---------- --CTCTCTTG CTTC------ -----TTGCT CTCTTGCTT- -CCTGCACCC

**IAP_10_180810**  TGCTCCTTAA GAGGGACGGG GTTT-TCGTT TTTCTCTCTC TCTTGC--TT CGCTCTCTTG CTTCGCTCTC TTGCTTCTCT CTCTTGCTT- -CTTGCTCTC

**IAP_11_180810**  TCTCTTTAAA GAGGAAAGGG GTTT-CG--T TTTCTCT--- ---------- --CTCTCTTG CTTC------ -----TTGCT CTCTTGCTT- -CTTGCACTC

**IAP_13_0180810**  TGCTCCTTAA GAGGGACGGG GTTT-CG--T TTTCTCT--- ---------- --CTCTCTTG CTTC------ -----TTGCC CTCTTGCTT- -CCTGCACCC

**IAP_14_180810**  TGCTCCTTAA GAGGGACGGG GTTT-CGTTT TCTCTCTCTC TTGCTTCT-- --CTCGCTCT TGCT------ ---TCTTGCT CTCTTGCTT- -CTTGCTCTC

**IAP_15_180810**  TGCTCCTTAA GAGGGACGGG GTTT-CGTTC TCTCTCTCTC TTGCTTCT-- --CTCGCTCT TGCT------ ---TCTTGCT CTCTTGCTT- -CTTGCTCTC

**IAP_18_180810**  TGCTCCTTAA GAGGGACGGG GTTT-CGTTT TCTCTCTTGC TT------CG CTCTCTCTTG CTTC------ -----TTGCT CTCTTGCTT- -CCTGCACCC

**IAP_19_180810**  TGCTCCTTAA GAGGGACGGG GTTT-TCGTT TTTCTCT--- ---------- --CTCTCTTG CTTC------ -----GCTCT CTCTTGCTT- -CTTGCTCTC

**IAP_20_180810**  TGCTCCTTAA GAGGGACGGG GTTTTCGTTT TCTCTCTCTC TTGCTTTTT- CGCTCTCTTG CTTT------ ---TCTCTCT CTCTTGCTTT TCTCTCTCTC

....|....| ....|....| ....|....| ....|....| ....|....| ....|..

210 220 230 240 250

**IAP_chromosome 17**  GGGCTCCTGA AGATGTAAGC AATAAAGTT- TTGCCGCAGA AGAT------ -------

**IAP_3_180810**  TTGCTCCTGA AGATGTAAGA AATAAAGCT- TTGCCGCAGA AGAT------ -------

**IAP_5_180810**  TTGCTTCTTG CTCTCTTGCT TCTTACACTC TTGCTCCTGA AGAT------ -------

**IAP_7_180810**  TGGCTCCTGA AGATGTAAGA AATAAAGCT- TTGCCGCAGA AGATTCTGGT CTGTGGT

**IAP_10_180810**  TT-TTCCTGA AGATGTAAGA A-TAAAGCT- TTGCCGCAGA AGAT------ -------

**IAP_11_180810**  TG-CTTCTGA AGATGTAAGA A-TAAAGCT- TTGCCGTAGA AGATTCTGGT CTGTGGT

**IAP_13_0180810**  TGGCTCCTGA AGATGTAAGA AATAAAGCT- TTGCCGCAGA AGATTCTGGT CTGTGGT

**IAP_14_180810**  TTGCTTCTTG CACTCTTGCT CCTGAAGATG TAAGCAATAA AG-------- -------

**IAP_15_180810**  TTGCTTCTTG CTCTCTTGCT TCTTGCACTC TGGCTCCTGA AGAT------ -------

**IAP_18_180810**  TGGCTCCTGA AGATGTAAGA AATAAAGCT- TTGCCGCAGA AGAT------ -------

**IAP_19_180810**  TT-TTCCTGA AGATGTAAGA A-TAAAGCT- TTGCCGCAGA AGATTCTGGT CTGTGGT

**IAP_20_180810**  TTGCTTCTTG CTCTCTTGCT TCTTGCTCTC TTGCTT---- ---------- -------
