# Supplementary material for: Mouse DNA contamination in human tissue tested for XMRV
Source: Retrovirology. 2010 Dec 20;7:108. doi: 10.1186/1742-4690-7-108 (PMC3019155; doi:10.1186/1742-4690-7-108)
Supplement: Supplementary file 3 [file 1742-4690-7-108-S2.DOC]

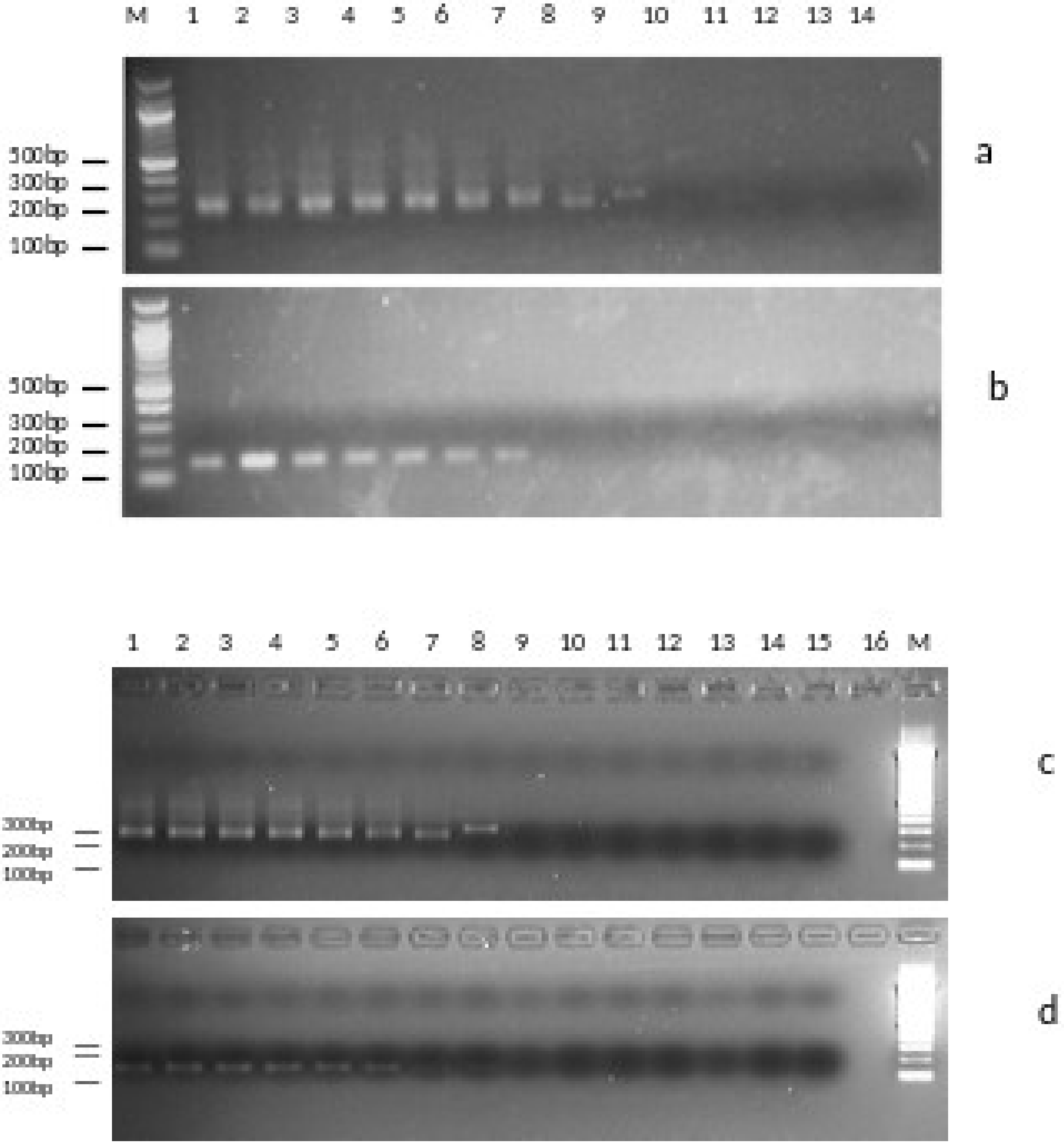
M 1 2 3 4 5 6 7 8 9 10 11 12 13 14
500bp
300bp
200bp
100bp
a
500bp
300bp
200bp
100bp
b
1 2 3 4 5 6 7 8 9 10 11 12 13 14 15 16 M
300bp
200bp
100bp
c
300bp
200bp
100bp
d
